# *Fugacium* Spliced Leader Genes Identified from Stranded RNA-Seq Datasets

**DOI:** 10.3390/microorganisms7060171

**Published:** 2019-06-11

**Authors:** Yue Song, Bahareh Zaheri, Min Liu, Sunil Kumar Sahu, Huan Liu, Wenbin Chen, Bo Song, David Morse

**Affiliations:** 1 Agricultural Genomics Institute at Shenzhen, Chinese Academy of Agricultural Sciences, Shenzhen 518124, China; 2 BGI-Qingdao, BGI-Shenzhen, Qingdao 266555, China; songyue@genomics.cn; 3 BGI-Shenzhen, Beishan Industrial Zone, Yantian District, Shenzhen 518083, China; liumin4@genomics.cn (M.L.); sunilkumarsahu@genomics.cn (S.K.S.); liuhuan@genomics.cn (H.L.); 4 State Key Laboratory of Agricultural Genomics, BGI-Shenzhen, Shenzhen 518083, China; 5 China National GeneBank, BGI-Shenzhen, Shenzhen 518120, China; 6 Institut de Recherche en Biologie Végétale, Département de Sciences Biologiques, Université de Montréal, Montréal, QC H1X 2B2, Canada; bahareh1987zaheri@gmail.com

**Keywords:** dinoflagellates, *Symbiodinium*, *Fugacium*, *trans*-splicing, spliced leader

## Abstract

*Trans*-splicing mechanisms have been documented in many lineages that are widely distributed phylogenetically, including dinoflagellates. The spliced leader (SL) sequence itself is conserved in dinoflagellates, although its gene sequences and arrangements have diversified within or across different species. In this study, we present 18 *Fugacium kawagutii* SL genes identified from stranded RNA-seq reads. These genes typically have a single SL but can contain several partial SLs with lengths ranging from 103 to 292 bp. Unexpectedly, we find the SL gene transcripts contain sequences upstream of the canonical SL, suggesting that generation of mature transcripts will require additional modifications following *trans*-splicing. We have also identified 13 SL-like genes whose expression levels and length are comparable to Dino-SL genes. Lastly, introns in these genes were identified and a new site for Sm-protein binding was proposed. Overall, this study provides a strategy for fast identification of SL genes and identifies new sequences of *F. kawagutii* SL genes to supplement our understanding of *trans*-splicing.

## 1. Introduction

**Figure 1:**
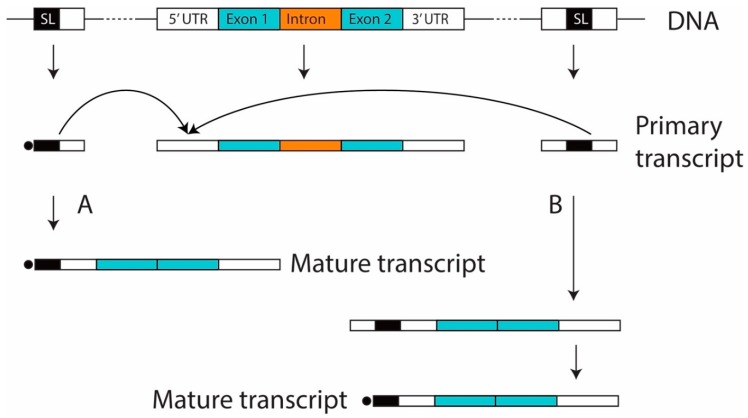
Spliced leader (SL) *trans*-splicing mechanism in dinoflagellates. (**A**) the mechanism previously thought; (**B**) a mechanism proposed based on the findings reported in this study, in which extra steps are needed to remove the sequences upstream from the SL before mRNA maturation.

Dinoflagellates are a large group of ecologically important unicellular algae. Many members in this lineage play critical roles in marine ecosystems as primary producers, contributors to red tides, and symbionts of reef corals and other invertebrates. Dinoflagellates are also known for their distinct genomic features which include large genome sizes, permanently condensed chromosomes, lack of nucleosomes [1]. Furthermore, the maturation of mRNAs in dinoflagellates has been proposed to require *trans*-splicing of a spliced leader (SL) sequence [2] (Figure 1A). 

The mechanism of *trans*-splicing had been reported in many other lineages including nematodes, flatworms, cnidarians, rotifers, chordates, and euglenozoans. The sequences of the spliced leader are conserved within each lineage but varies among different groups. Different roles of *trans*-splicing have been proposed, which include translation regulation (enhancing or blocking), mRNA stabilization, 5′UTR sanitization, protein retargeting, as well as creating or destroying upstream open reading frames [3,4,5]. Several lineages have more than one consensus SL sequence; for example, euglenozoans can have as many as 14 SL sequences [6]. On the other hand, dinoflagellates have only one, (DCCGUAGCCAUUUUGGCUCAAG (D = U, A or G)) [2]. Attempts to clone SL genes have been made using *Prorocentrum minimum*, *Karenia brevis*, *Polarella glacialis*, *Heterocapsa arctica*, *Karlodinium veneficum*, and *Pfiesteria piscicida* [2,7]. Despite the conservation of SL within this phylum, the arrangements of SL RNA genes are rather diverse. In these organisms, SL genes were clustered alone or mixed with 5S rRNA. The introns in these genes also showed substantial differences.

The presence of SL on the 5′ end of all mature mRNAs has greatly advanced research on dinoflagellates, particularly for Symbiodiniaceae, a group of symbiotic dinoflagellates, by facilitating the identification of dinoflagellate transcripts from mixed samples [8] and identification of retrogenes in the transcriptome [9,10,11] and the genome [12]. However, the SL RNA genes themselves had still not been cloned in Symbiodiniaceae even though several genome assemblies had been released [13,14,15,16]. Previous attempts of SL RNA gene cloning relied on polymerase chain reaction with a SL-derived forward primer, which limited the identification of sequences upstream of SLs. High-throughput sequencing of cDNA libraries provided an alternative approach for the identification of SL transcripts, but without strand information there would be many mistakes and uncertainties. This problem can be solved by strand-specific sequencing, in which the 5′ terminus of transcripts will be certainly found in the forward or reverse reads depending on the strategy of library construction [17]. 

In this study, we identified 18 SL and 13 SL-like genes from stranded RNA-seq reads, and found extra sequences upstream of SLs. The SL genes identified in this study are generally longer than those previously reported. Introns from these genes are also identified and a new potential site of Sm-protein binding is proposed. 

## 2. Materials and Methods

### 2.1. Genomic and Transcriptomic Data

The datasets of stranded RNA-seq reads of *F. kawagutii* (previously called *Symbiodinium kawagutii*) [18] were downloaded from NCBI under the accessions of SRP182908 and SRP119222 [19]. Previously unpublished transcriptome sequences were prepared from TRIzol-purified RNA samples taken at two times during a 12:12 light/dark cycle (lights on and lights off). Quality control, TruSeq stranded mRNA sample preparation (including poly(A) RNA purification), and Illumina sequencing using a HiSeq 4000 was performed at the McGill University and Genome Quebec Innovation Centre (Montreal, Canada). The draft genome sequences of *F. kawagutii* [14] were also downloaded from NCBI.

### 2.2. Clustering and Assembly of SL-Containing Reads

SL-containing reads were identified by searching for SL sequences (CCGTAGCCATTTTGGCTCAAG) in the reverse reads (R2). Clustering and assembly were performed by iAssembler (version v1.3.3) software [20] with a minimum overlap of length >30 bp and identity >95%.

### 2.3. Identification of Sm-Protein Binding Sites and Structural Analysis of SL Genes

Five U-rich motifs in introns of Dino-SL genes were selected and their appearances in the introns of *F. kawagutii* genes were counted and normalized to their expected appearance as a random occurrence (1/n^4^, where n is the length of motif). The secondary structure of SL genes was simulated using MFOLD online service (http://unafold.rna.albany.edu/?q=mfold) with the folding temperature set at 20 °C [7].

## 3. Results

### 3.1. Searches for SL-Containing Reads

**Figure 2:**
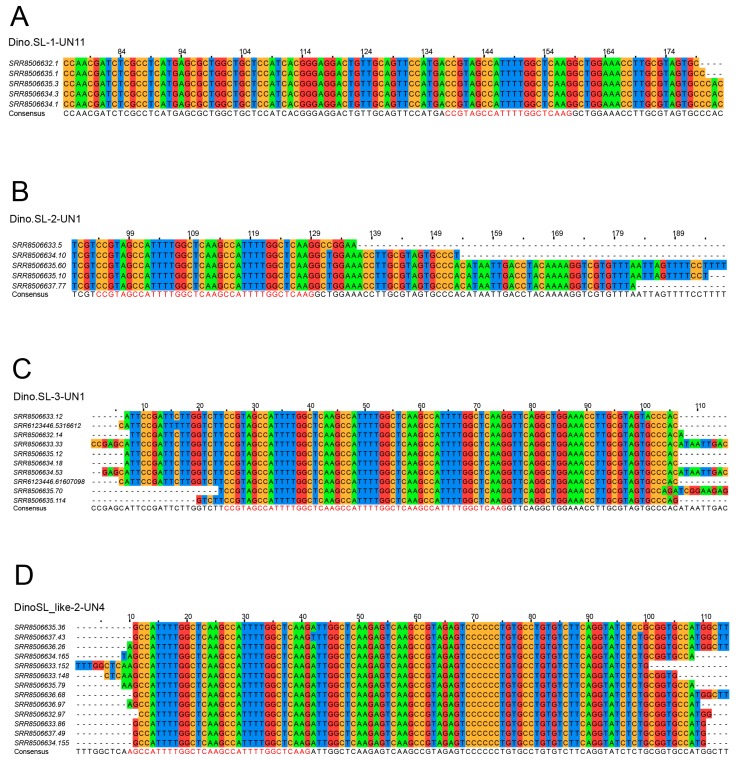
Alignment and assembly of SL-containing reads. The consensus sequence is shown at the bottom of each alignment. Examples include (**A**) single SL genes, and tandem SL genes with (**B**) two and (**C**) three units, and (**D**) an example of SL-like genes. The sequences of SL and its relicts were colored in red in the consensus sequences. More examples can be found in the Supplementary Figure S1.

We identified SL-containing reads by searching for SL sequences in the reverse reads (R2) in the stranded RNA-seq datasets. In total, we obtained 195 SL-containing reads from 8 libraries constructed for this study and from a previously published dataset [19]. Among these, 154, 31, and 10 contained one, two, and three units of SL, respectively. We also found several SL-containing genes that also contained SL relicts (AGCCATTTTGGCTCAAG) (Supplementary Figure S1). The sequences were clustered into 18 groups according to their similarities, and their consensus sequences were obtained by aligning and assembling the sequences in each group (Supplementary Table S1). These sequences are thus likely to be derived from SL genes. There are 16 sequences with a single copy of the SL, one with 2 SL units and one with 3 SL units (Figure 2; Supplementary Table S1). Remarkably, we note the presence of sequence upstream from the canonical SL which, after *trans*-splicing, constitutes the 5′ end of all dinoflagellate mRNA, suggesting extra steps are required to remove these sequences before mRNA maturation (Figure 1B).

### 3.2. Tandem-SL Genes

Among the reads containing both SL and multiple SL relicts (AGCCATTTTGGCTCAAG), 31 have two SLs and 10 have three SLs. Interestingly, reads containing two SLs clustered into one group while reads with three SLs clustered in another (Supplementary Table S1). We noticed that unlike the sequence diversity observed downstream of SL in reads with only a single SL (Supplementary Figure S1), the 3′ downstream sequences in these reads were conserved (Figure 2). These similar/identical reads were not the products of PCR duplicates in the libraries because duplicates had already been removed before the analysis. Moreover, these reads were found in different libraries, both in this study and in a previously reported work [19]. One possible explanation is that they were from highly expressed genes, which would have a greater chance of being modified by addition of multiple SLs [12,21]. However, we excluded this possibility because they were also found in the genome assembly of *F. kawagutii*. Therefore, these different consensus sequences represent different loci of tandem SL.

### 3.3. Introns in SL Genes 

We identified 18 types of introns from the different SL loci. In the loci of single SL genes, 16 different sequences (Supplementary Table S1) were found after the 3′ end of SL (AAG). In the loci of tandem SL, two types of different sequences downstream of SL were found (Supplementary Table S1 and Figure S2). All these downstream sequences started with GU or GC, the motifs characteristic of the 5′ end of dinoflagellate introns [13]. 

**Figure 3:**
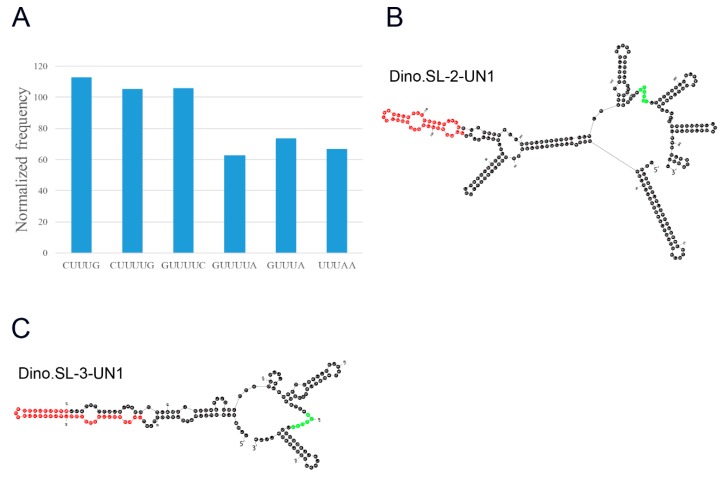
Potential Sm-protein binding sites. (**A**) The appearances of U-rich motifs in introns of *F. kawagutii* genes; the secondary structure of SL genes, (**B**) Dino.SL-2-UN1, (**C**) Dino.SL-3-UN1. The proposed Sm-protein sites (GUUUUC) were colored in green and the sequences of SL were colored in red in (**B**,**C**). The sequences of these SL genes can be found in the Supplementary Table S1.

Sm-protein binding sites, which always have oligo(U) motifs, are conserved in different organisms [22,23,24], and oligo(U) presence constitutes evidence of an intron. We have analyzed the sequences of these introns to identify the potential Sm-binding sites. Previous works proposed that the Sm-protein binding site was the AUUUUGG located in the SL exon, instead of being found in the intron [2]. However, this seems unlikely given that Sm-protein binding sites are usually located in introns—indeed, there is no precedent as yet for Sm-protein binding to exons. We found reads with several U-rich motifs (CUUUUG, GUUUA, GUUUUC, GUUUA, GUUUUA, and UUUAA) in the introns. None of these motifs was identical to the known Sm-protein binding sites. These results suggest that the Sm-protein binding sites in dinoflagellates may be different from any of the sites known in other organisms. We further counted the appearances of these motifs in the introns of *F. kawagutii* genes and found that CUUUG, CUUUUG, and GUUUUC are more frequent in introns (Figure 3A). We then examined their location in the predicted RNA structure of the SL gene transcripts. Interestingly, despite differences in sequences and length, the different structures share some conserved features. In particular, the SL sequence is found in the stalk of a “Y” shape structure formed from three stem–loops (Figure 3B,C). A bulge, a feature of Sm-protein binding sites, appeared at a GUUUUC motif in the fork of the Y in the SL transcript. Therefore, we propose GUUUUC as a potential Sm-protein binding site in *F. kawagutii*. 

### 3.4. SL-like Genes

Besides these SL loci with single or tandem SLs, we also found several reads bearing multiple repeats of SL relicts (AGCCATTTTGGCTCAAG). For example, there was a transcript having three tandem SL relicts but from which no bone fide SL could be recovered. Aligning and assembling of these sequences resulted in 13 consensus sequences (Supplementary Table S1). We inspected these sequences closely and found a minor difference at the start of the first unit of SL relict, in which the TCCG of a canonical SL was replaced by TCG (Figure 2). This is unlikely to be a sequence error because these sequences were repeatedly found in 8 different independently constructed libraries. We also confirmed that these reads corresponded to sequences found in the genome. Although the sequences at these loci are different from loci containing SL, the relict sequences were also transcribed at a higher level (17 reads/million) than the authentic SL gene sequences (4.6 reads/million). It is possible that these SL-like loci are located near or at the same cluster of SL loci and shared the same mechanism of transcription and regulation. However, they may not be functional because, unlike the identified SL loci, intron donor sites (G^U^/_C_) and putative Sm-protein binding sites (GUUUUC) were absent from the sequences downstream of tandem SLs (Figure 2; Supplementary Table S1). This suggests that SL or its relicts may not be able to be spliced from these loci.

## 4. Discussion

Despite being found in numerous species, the evolution of *trans*-splicing machinery and SL genes are enigmatic. In dinoflagellates, several SL genes have been cloned in species ranging from *Polarella* and *Heterocapsa* to *Prorocentrum* and *Pfiesteria* [2,7]. As shown by these reported SL genes, the sequences and genomic organizations of SL genes are diverse [7]. Therefore, more information about these SL genes—particularly those in species other than those aforementioned—is needed to further our understanding of the character and evolution of these genes. Indeed, the *F. kawagutii* SL genes identified in this study displayed several features different from those previously reported. 

### 4.1. The Lengths of SL Genes in F. kawagutii Are Longer

The lengths of SL genes identified in this study range from 103 to 292 bp, with an average of 164 bp (Supplementary Table S1), which is remarkably longer than those reported in *P. minimum*, *K. brevis*, *P. glacialis*, *H. arctica*, *K. veneficum*, and *P. piscicida*, which are predominantly 50–60 bp in length [7]. In previous studies [2,7], SL genes had been cloned using a 3′ RACE strategy with SL-derived forward primers, assuming SL was the 5′ terminus of each unit. As a consequence, the sequences upstream of SL were undetectable. Therefore, the lengths of SL genes may have been underestimated in previous studies. We also remeasured the length of *F. kawagutii* SL genes excluding these upstream sequences. However, the lengths of *F. kawagutii* SL gene averaged to 89 bp, which is still longer than those reported. As these *F. kawagutii* SL genes were identified using single-ended or pair-ended stranded reads from libraries with insertion sizes of ~200 bp [19], the lengths of these SL genes may have also been underestimated. Transcript assembling may partially mitigate this problem but may also possibly introduce errors by, for example, linking reads derived from different loci.

### 4.2. The Fate of the Upstream Sequences

It has been generally assumed that the canonical SL sequence was located at the 5′ end of the transcript derived from the SL gene. Since these transcripts were also thought to be capped at their 5′ end, the *trans*-splicing mechanism was thus responsible for providing the cap structure on all other transcripts. However, the presence of sequence upstream of the SL as shown here suggests the generation of mature transcripts may be more complicated than previously thought (Figure 1B). The sequences immediately upstream of the SL do not show any conserved features in the different versions of the SL gene transcript, yet these must clearly be removed as no transcripts have yet been detected in any dinoflagellate transcriptome to date with sequence upstream from the SL. Lastly, a mechanism must have evolved for capping the transcripts after the excess sequences have been removed. These upstream sequences were not included in the previously cloned SL genes because they were cloned using 3′ RACE strategy with SL-derived forward primers; SL was assumed to be the 5′ terminus of each unit during design of this cloning strategy [2,7].

### 4.3. Novel Sm-Protein Binding Sites

The presence of Sm-protein binding sites, which are usually found in introns of genes in various organisms, is in fact a criterion used for the determination of intronic sequences of genes. Sm-protein binding sites were also found in introns of various SL genes in different organisms [6]. Dinoflagellates constitute an exception in that canonical Sm-protein binding sites (AUUUUGG) were found in the exons of SL genes [2,7]. Given that the Sm-protein binding sites would normally be spliced off, blocking further Sm protein binding, its presence in exons is puzzling. In this work, we found a U-rich motif, GUUUUC, which was found in the introns of the identified SL genes in *F. kawagutii*. We have therefore proposed this sequence—which is different from the one proposed in other studies [2,7]—as a potential Sm-protein binding site. One possible reason for the differences may lie in the fact that the species studied here (*F. kawagutii*) was different from those used in previous studies (*P. minimum*, *K. brevis*, *P. glacialis*, *H. arctica*, *K. veneficum*, and *P. piscicida*). Another explanation could be the different strategies used for SL gene cloning in different works. In previous studies, SL genes were cloned using RACE techniques from non-poly(A) RNA, which after removal of poly(A) containing transcripts, were ligated with oligo(A) before reverse transcription [2,7]. During this procedure, the oligo(A) tail might have been ligated with trimmed or fragmented transcripts leading to cloning of partial length SL genes. The 3′ regions bearing the true Sm-protein binding sites might thus have been missed if only the 5′ part of the SL genes were cloned. Lastly, different fractions of transcripts were selected for analysis in this compared to previous studies. SL genes cloned using 3′ RACE were derived from non-poly(A) transcripts while those identified from stranded RNA datasets in this study were derived from polyadenylated transcripts. However, we cannot know with certainty that the transcripts sequenced in this study were polyadenylated, as some residual fraction of unmodified RNA may still be present after the poly(A) purification step. 

### 4.4. SL-like Genes in F. kawagutii

We also identified 13 SL-like genes in *F. kawagutii*. All of them have multiple full or partial SLs. This is due to the fact that candidates with only one SL or its relict were removed to exclude the possibility of false discoveries caused by random appearance of the SL sequence. This would thus lead to a failure to identify any SL-like genes with only one SL unit. These genes recovered are very similar in sequences to the real SL genes, but differ in that the donor sites for intron splicing are lost. Furthermore, they also lack Sm-protein binding sites. We speculate that these genes cannot be accurately spliced and represent pseudo-SL genes. According to a rough estimation of their expression levels based on their read counts in the different libraries, the expression levels of these SL-like genes are comparable to that of real SL genes. 

### 4.5. Tandem-SL Genes in F. kawagutii

Multiple tandem SLs or their relicts had been found in transcripts of many genes of various organisms ranging from *Perkinsus marinus* and *Oxyrrhis marina* to *Alexandrium tamarense* [9,10]. The appearance of relicts was interpreted as an accumulation resulted from multiple rounds of *trans*-splicing during the evolution of dinoflagellates. It is interesting that all SL relicts found in these transcripts start with “CCA”—they are thus missing the upstream CCGTAG of SL. One possibility to account for this is that CCGTAG may have been spliced off, given that “AG” could provide a site for intron splicing. However, the SL relicts identified in genomes are longer and contain CCGTAG. The sequence of the tandem partial SLs in the SL genes suggests an alternative interpretation of their appearance in transcripts: there was only one *trans*-splicing event derived from a tandem-SL gene, instead of multiple rounds of *trans*-splicing from single copy SL transcripts.

Overall, we identified 18 SL genes including 16 single and 2 multiple-SL genes, as well as 13 multiple SL-like genes which have degenerated due to the loss of intron splicing donors. This study provides important supplements to our knowledge of SL genes and illustrates how SL genes can be identified from stranded RNA-seq datasets. The number of SL genes identified in this work is rather limited and presumably only represents a small fraction of the SL genes in *F. kawagutii* genome, at least in part because the datasets used were obtained from polyadenylated transcripts which are likely to be underrepresented in SL genes. Nevertheless, the success identification of SL genes suggests stranded RNA sequencing is a feasible and efficient approach for SL gene identification. 

## Supplementary Materials

Supplementary materials can be found at https://www.mdpi.com/2076-2607/7/6/171/s1.
