# Supplementary material for: Fugacium Spliced Leader Genes Identified from Stranded RNA-Seq Datasets
Source: Microorganisms. 2019 Jun 11;7(6):171. doi: 10.3390/microorganisms7060171 (PMC6616646; doi:10.3390/microorganisms7060171)
Supplement: Supplementary file 1 [file microorganisms-07-00171-s001.zip › Supplementary figure 1.pdf]

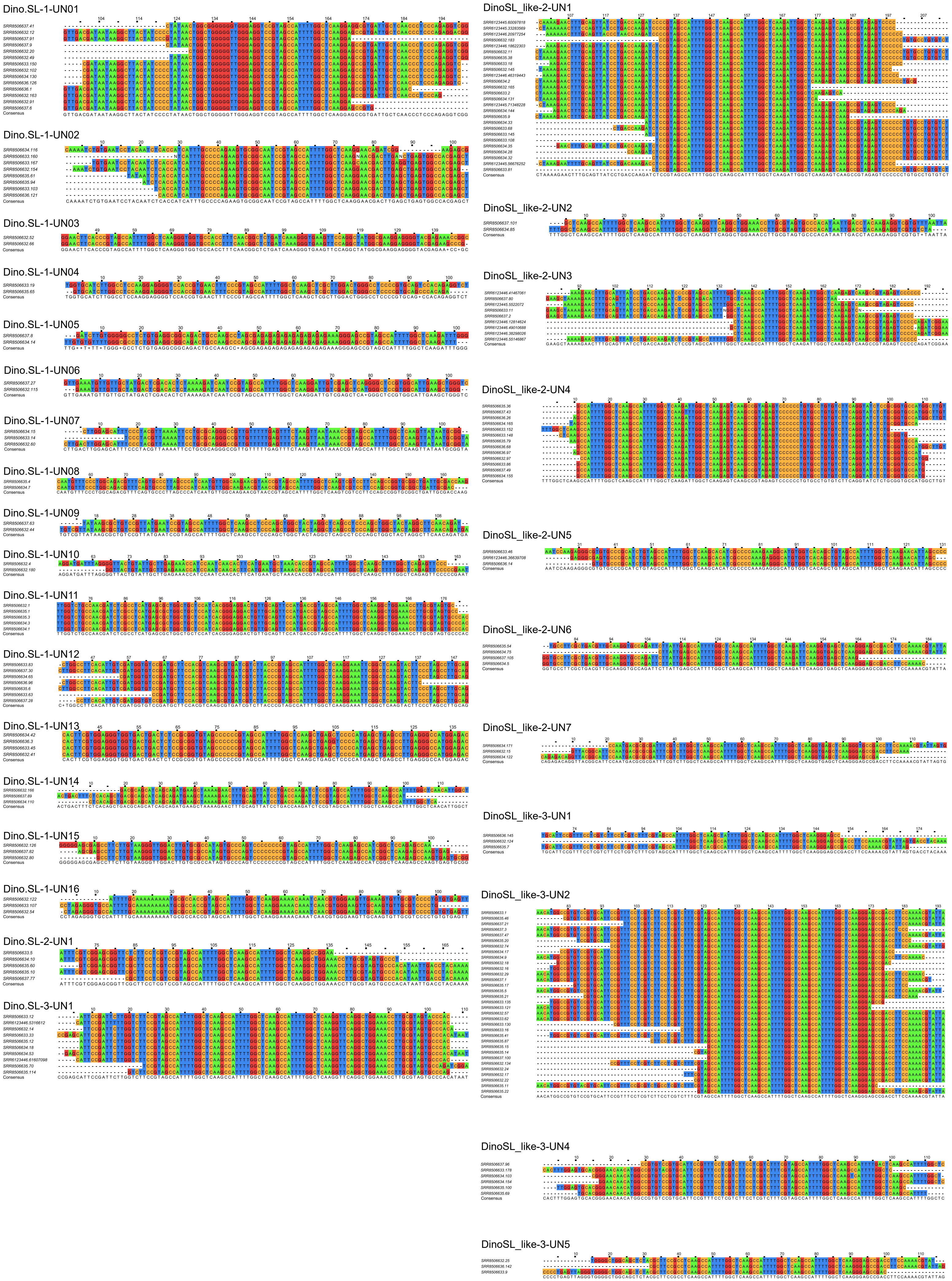
Dino.SL-1-UN01
Dino.SL-1-UN02
Dino.SL-1-UN03
Dino.SL-1-UN04
Dino.SL-1-UN05
Dino.SL-1-UN06
Dino.SL-1-UN07
Dino.SL-1-UN08
Dino.SL-1-UN09
Dino.SL-1-UN10
Dino.SL-1-UN11
Dino.SL-1-UN12
Dino.SL-1-UN13
Dino.SL-1-UN14
Dino.SL-1-UN15
Dino.SL-1-UN16
Dino.SL-2-UN1
Dino.SL-3-UN1
DinoSL_like-2-UN1
DinoSL_like-2-UN2
DinoSL_like-2-UN3
DinoSL_like-2-UN4
DinoSL_like-2-UN5
DinoSL_like-2-UN6
DinoSL_like-2-UN7
DinoSL_like-3-UN1
DinoSL_like-3-UN2
DinoSL_like-3-UN4
DinoSL_like-3-UN5
